# Ethylene as the Molecular Coordinator of the Plant Growth–Defense Trade-Off Under Biotic and Abiotic Stresses

**DOI:** 10.3390/ijms27125576

**Published:** 2026-06-20

**Authors:** Md. Rasel Mia, Abira Sahu, Mrinmoy Kundu, Md. Ejaj Uddin Khan, Monisha Akter Rupa, Farjana Sultana, Mohammad Golam Mostofa, Md. Motaher Hossain

**Affiliations:** 1 Department of Plant Pathology, Gazipur Agricultural University, Gazipur 1706, Bangladesh; raselmia.gau@gmail.com (M.R.M.); mrinmoykundu36@gmail.com (M.K.); mdejajuddinkhan99@gmail.com (M.E.U.K.); monisharupa86@gmail.com (M.A.R.); 2 Department of Environmental Health Sciences, University of Alabama at Birmingham, Birmingham, AL 35233, USA; sahua@uab.edu; 3 College of Agricultural Sciences, International University of Business Agricultural and Technology, Dhaka 1230, Bangladesh; farjana1s@iubat.edu; 4 Department of Chemistry, State University of New York College of Environmental Science and Forestry, Syracuse, NY 13210, USA

**Keywords:** climate-resilient crops, ethylene signaling, growth–defense trade-off, hormonal crosstalk, plant stress responses, transcriptional networks

## Abstract

Plants must continuously balance the trade-offs between growth and defense, a constraint that is exacerbated by biotic and abiotic stresses, particularly when they occur together. Ethylene (ET) serves as a central, integrative regulatory node controlling this by linking developmental programs to stress-responsive signaling networks. Advances at the molecular and systems levels have revealed that ET mediates the redistribution of metabolic resources via coordinated regulation of its synthesis, perception, and downstream signaling. The ETR (Ethylene Receptor)-CTR1 (Constitutive Triple Response 1)-EIN2 (Ethylene Insensitive 2)-EIN3(Ethylene Insensitive 3) signaling module lies at the core of this network, integrating multiple hormonal pathways. Through dynamic crosstalk with jasmonic acid (JA), salicylic acid (SA), abscisic acid (ABA), auxin (AUX), and gibberellins (GA), ET enables the fine-tuned coordination of growth inhibition, immune activation, and stress acclimation in response to environmental fluctuations. Processes such as induced systemic resistance, programmed cell death, and architectural plasticity further reinforce this regulatory framework, with ethylene-responsive transcription factors, including ERFs (ethylene responsive factor gene family) and WRKYs, acting as critical convergence points. Emerging insights into ACC (1-aminocyclopropane-1-carboxylic acid)-dependent signaling, chromatin remodeling, and tissue-specific regulation expand the functional scope of ET beyond traditional hormone paradigms. At the same time, the ability of pathogens to manipulate ET signaling underscores its dual role in both promoting immunity and facilitating susceptibility. By integrating molecular, physiological, and ecological perspectives, this review highlights ET as a central coordinator of plant stress resilience and growth optimization, providing a unifying framework for understanding how plants adapt to complex and dynamic environments.

## 1. Introduction

Plants continually strive to balance between their growth and defense responses, particularly under adverse environmental conditions. As sessile organisms that cannot escape unfavorable conditions, plants have evolved intricate molecular mechanisms to reallocate resources from growth and differentiation toward protection and survival. This growth–defense trade-off involves complex regulatory networks governed by transcriptional, hormonal, and metabolic mechanisms that integrate development, immunity, and adaptation in plants [1,2,3]. Activation of defense responses is resource-intensive, requiring the diversion of carbon (C) and nitrogen (N) pools from biomass accumulation to the synthesis of protective metabolites. This often results in reduced growth rates and remodeling of developmental programs [1]. At the regulatory level, small RNAs, including microRNAs, and transcription factors such as WRKY function as key nodes that coordinate hormonal and transcriptional crosstalk, linking AUX-mediated growth with defense pathways driven by JAand SA [1,2]. Other signaling molecules, such as nitric oxide (NO), heterotrimeric G-proteins, and redox intermediates, integrate phytohormone signaling pathways, particularly those involving ABAand SA, with stress responses and developmental transitions [4,5].

At the metabolic level, stress conditions trigger extensive reprogramming of primary metabolism, channeling energy and resources toward the production of defense-related secondary metabolites. For example, in potato, a decline in relative growth rates under biotic stress has been shown to coincide with the induction of secondary metabolism [6]. Similarly, regulatory modules such as the OsSGS3-tasiRNA-OsARF3 pathway in rice exemplify how plants integrate multiple stress signals to balance competing demands, coordinating heat tolerance with pathogen defense [7]. Within this intricate network, ET emerges as a central signaling hub that modulates a wide array of developmental and stress-related processes, including fruit ripening, leaf abscission, seed germination, and stress acclimation [8,9]. ET often plays a pivotal role in maintaining plant growth and fitness under stress. ET regulates stomatal closure under drought, modulates ROS scavenging under salinity, and coordinates elongation responses during flooding via crosstalk with GA [10,11]. Plant responses to temperature stresses (cold and heat) often involve distinct yet sometimes overlapping molecular and physiological pathways modulated by ET [12,13]. In biotic interaction, ET acts as master regulator of immune responses against diverse pathogens and insect herbivores [14,15,16]. Depending on the nature of the biotic stress and the developmental context, ET can either promote or demote defense responses, thereby optimizing resource allocation between survival and productivity [17]. Conversely, pathogens can also manipulate ET biosynthesis and signaling to promote susceptibility, underscoring its dual role in immunity and disease facilitation [18,19].

Interestingly, ET modulates the trade-off between resource allocation and growth–defense by mediating crosstalk among SA, AUX, GA, JA, and ABA [20,21]. Understanding the molecular harmonization by which these integrative systems operate provides critical insights into ET-mediated molecular mechanisms that support plant adaptation, tolerance, and growth upon environmental perturbations [22,23].

In this review, we examine the multifaceted roles of ET as a central molecular regulator of the growth–defense balance in plants under stress. The molecular mechanisms underlying environmentally induced ET biosynthesis are discussed. We also highlight ET-mediated regulation of key developmental processes such as cell expansion, organ morphogenesis, senescence, flowering, and fruit ripening. Collectively, these perspectives illustrate how ET integrates developmental and stress-responsive signaling networks, highlighting its pivotal role in plant adaptation in dynamic environments.

## 2. Conceptual Framework: The Growth–Defense Trade-Off

### 2.1. The Growth–Defense Dilemma: Metabolic and Energetic Constraints

The growth–defense trade-off represents one of the most fundamental challenges in plants, arising from the need to allocate finite metabolic and energetic resources between biomass production and immunity [24]. A widely recognized conceptual framework for understanding this balance is the Growth-Differentiation Balance (GDB) hypothesis, which posits that growth and defense are inherently constrained by competition for shared limiting resources within plant cells and tissues. Consequently, enhanced investment in defensive differentiation is often associated with reduced growth capacity, reflecting a core trade-off that shapes plant physiological and ecological strategies [25].

At the cellular level, prioritization of growth supports rapid biomass accumulation but typically occurs at the expense of defense-related differentiation. In contrast, activation of defense pathways entails substantial metabolic costs, including the biosynthesis of carbon (C)-, nitrogen (N)-, and sulfur (S)-containing secondary metabolites, as well as structural reinforcements, all of which can restrict resource availability for growth [24]. Such opposing constraints are particularly evident under fluctuating environmental conditions. For example, under water stress, plants accumulate higher levels of C-based secondary metabolites in leaves while reducing their level in roots, highlighting tissue-selective metabolic reprogramming toward defense rather than growth [26].

**Figure 1:**
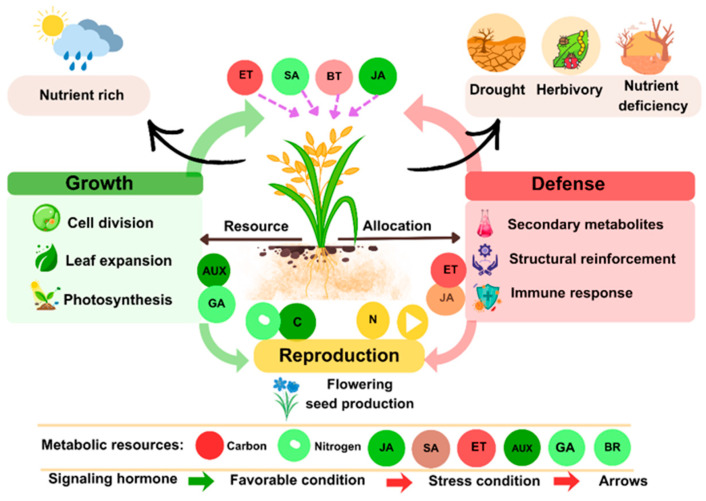
A conceptual framework explaining the growth–defense trade-off in plants in diverse environments. The scheme represents the differential investment of metabolic resources (C and N) along with signaling hormones (e.g., JA, SA, ET, AUX, and GA) in physiological growth programs, including cell division processes and photosynthesis-supported growth processes, as well as defensive responses towards various biotic and abiotic stress. The shift in focus of resource allocation is represented by arrows; favorable nutrient-rich regimes favor vegetative growth and reproduction, whereas stress conditions, such as drought, herbivory and limited nutrients, favor defensive responses that include the production of secondary compounds as well as structural reinforcement. This model emphasizes the trade-offs in plants to increase survival and fitness in fluctuating environments.

At the molecular level, this balance is governed by key regulatory genes. In rice, coupling the growth morphology with immunity was mediated by aldehyde dehydrogenase (*OsALDH2B1*), which interacts with brassinosteroids (BRs), G-proteins, JA, and SA signaling pathways [27]. Mutations that reduce expression of these genes enhance defense responses but impose significant penalties on growth, fertility, and yield, providing evidence for tight coupling between gene-regulatory phenotypes and metabolic resource allocation [27] (Figure 1).

Ecologically and evolutionarily, this trade-off has implications for genetic variation and adaptation. Variation in allocation strategies among genotypes reflects adaptation to environmental contexts, with fast-growing plants typically investing less in defense under resource-rich or low-herbivory conditions [28]. These strategies influence not only individual fitness but also community dynamics and broader ecosystem functioning. To mitigate the costs associated with defense, plants have evolved a range of adaptive strategies such as sequestering toxic metabolites in vacuoles, recycling secondary compounds into primary metabolism, and inducible defense mechanisms that are activated only upon damage [24]. Additionally, phenotypic plasticity enables adaptive reallocation of investments in response to environmental cues, favoring growth under high-nutrient conditions and enhancing defense during stress or resource limitation [29]. Collectively, the growth–defense trade-off mechanisms are regulated across multiple levels by complex interactions among hormonal, genetic, and environmental networks.

### 2.2. Hormonal Cross-Regulation as the Decision-Making System

Hormonal crosstalk constitutes a central regulatory framework through which plants integrate growth and defense programs in response to dynamic environmental and developmental cues. This multilayered network coordinates phytohormone signaling with transcriptional regulation and small RNA-mediated control to achieve metabolic and physiological balance. Among these, microRNAs (miRNAs) play a pivotal role in fine-tuning hormone interactions, linking growth and defense responses, and providing promising targets for engineering stress-resistant and high-yielding crops [1]. Additionally, a set of five genetically linked effectors, called Topless (TPL)-interacting protein (Tip) effectors, was identified. These effectors induce AUX signaling by interfering with the central corepressors of the TPL family. This finding reveals that TPL proteins, which are key regulators of growth–defense antagonism, are major targets of the *Ustilago maydis* effectome [30]. Within this interconnected framework, ET represents a versatile regulator that coordinates plant responses to abiotic stress through the canonical ETR-CTR1-EIN2-EIN3/EIL1 signaling cascade. It is integrated with other hormonal pathways, particularly ABA and AUX, that collectively govern growth–stress trade-offs [10] (Table 1). In this framework, ET functions as a hormonal hub, where, upon stress sensing, biosynthetic and signaling pathways are activated to rapidly stimulate defense-related secondary metabolism and repress growth-associated gene expression. The crosstalk between ET and growth-promoting hormones GA and AUX makes ET an important regulatory node for the balance between development and defense, as well as for energy-efficient adaptation in plants [31].

These hormonal interactions are further modulated by environmental inputs, including nutrient availability, particularly C and N status, which influence the extent to which plants prioritize growth or defense [29]. External cues such as light quality also intersect with hormonal networks; for instance, shade-sensing photoreceptors modulate JA signaling to attenuate defense responses under competitive conditions, reflecting an integration of sensory perception with hormonal regulation [32].

**Table 1:** Summary of phytohormone signaling modules and their integrated roles in balancing plant growth–defense trade-offs and environmental stress resilience.

Hormone	Principal Growth-Related Roles	Defense-Associated Roles	Core Signaling Components	Hormonal Crosstalk and Trade-Off Implications	Representative Phenotypic or Molecular Evidence	References
ET	Growth regulator; balances growth-stress	Broad abiotic-stress responses	ACS/ACO; ETRs-CTR1-EIN2-EIN3/EIL1; EBF1/2	Hub crosstalk with ABA/JA/BR/AUX/GA/SA/CK; tolerance can reduce growth	ET as key for growth-stress trade-offs	[10]
GA	Growth/elongation	Flooding adaptation	GID1; DELLA; GA20ox/GA3ox; GID2/SLY1	ET can promote or suppress GA elongation depending on submergence depth	Flooding model: ET → *SK1/2* or *SUB1A* → DELLAs controls elongation	[10]
ABA	Growth inhibition	Stomatal closure, drought/osmotic tolerance	PP2Cs-SnRKs; ABI4; MHZ4/5	Often antagonistic with ET; ABA can repress ACS (ABI4) while ET can raise ABA via MHZ4/5	ABA-ET model in guard cells (ROS/NO, closure control)	[10]
SA	Defense activation can reduce growth/seed production (defense-growth trade-off)	Generally effective against biotrophic pathogens	*NPR1* → activation of defense genes including *PR1* marker	Antagonizes JA: JA genes (e.g., *PDF1.2*, *VSP2*) sensitive to suppression by SA; can create ecological cost (higher susceptibility to necrotrophs) but may be cost-effective overall	SA (or SA-inducing *H. arabidopsidis*) suppresses *B. cinerea-induced PDF1.2*, increasing susceptibility SA/MeJA induces *PR1*, and SA/MeJA suppresses *VSP2* vs. MeJA alone	[33]
JA (*ERF*-branch; JA/ET-associated)	JA defense activation can cause fitness costs (reduced growth/seed; delayed flowering)	Generally effective against necrotrophic pathogens	*ERF*-branch regulated by ERF1, ORA59, co-regulated by ET, marker *PDF1.2*	*ERF*-branch is suppressed when SA pathway or *MYC*-branch is activated first → lower resistance to necrotroph (*B. cinerea*)	*B. cinerea* induces *PDF1.2*, but it is strongly repressed after prior SA-induction or *P. rapae* (*MYC*) induction; induced plants become more susceptible to *B. cinerea*	[33]
AUX (IAA)	Major growth/development hormone (trade-off partner with JA).	Can be inversely related to JA-mediated defense strength via shared machinery.	Shared reliance with JA on SCF-type ubiquitin E3 complexes; wound-inducible amidohydrolases (IAH) tune JA-Ile + IAA.	Shared components can shift balance: AUX recruitment of shared SCF components may limit JA defense, and vice versa.	*arx1/arx6* mutants: reduced AUX response and reduced JA sensitivity.	[31]
JA	Regulates development; can inhibit growth when defense is activated.	Broad defense against necrotrophs, herbivores, and viruses.	JA-Ile → COI1 (SCF^COI1 with ASK1/CUL1) → *JAZ* degradation → MYC TF activation; repression via NINJA/TPL.	Central hub balancing growth/defense and interacting with SA, ET, GA, AUX, phytochrome.	*jazD* (*10 JAZ genes*) = strong resistance but slow growth/poor fertility, showing growth-immunity antagonism.	[31]
BRs	Strong growth promotion (growth-promoting steroids perceived at plasma membrane)	Can modulate/inhibit immune signaling	*BRI1* receptor + co-receptor BAK1/SERK3	Shared co-receptor (BAK1) links growth and PTI; BR perception can suppress immune outputs (BAK1-dependent and independent)	BR perception inhibits FLS2-mediated immunity; BRI1 overexpression reduces BAK1-dependent immune responses	[34]

### 2.3. Positioning ET Within the Trade-Off Framework

ET plays a central role in balancing between plant growth and defense, mediating both hormonal crosstalk and environmental signal integration. ET acts in concert with JA, SA, ABA, AUX, GA, and cytokinin (CK) to balance growth-promotion with defense-activation along a dynamic continuum [10,29]. In maize, ET interacts with JA and ABA pathways to regulate developmental transitions and stress-induced defense metabolism, contributing to yield stability under adverse conditions [35].

At the molecular level, ET signaling is coordinated by the transcription factor ETH-YLENE-INSENSITIVE3 (EIN3)/EIL that acts in coordination with *MYC2*-regulated complexes to mediate the defense response against insects and pathogens, a typical example of hormonal crosstalk in plants [36]. Moreover, the ET precursor 1-aminocyclopropane-1-carboxylic acid (ACC) can act independently of canonical ET receptors (ETR1/ERS family) or the downstream CTR1–EIN2–EIN3 module to influence growth and defense in specific developmental and stress contexts [37]. Instead, it may act through yet unidentified plasma membrane-associated transport/signaling systems and downstream transcriptional regulators that are distinct from classical ET signaling components. Epigenetic mechanisms also contribute to this regulatory network, with chromatin modifications such as histone acetylation modulating the transcriptional responsiveness of ET-regulated genes [38]. Functionally, ET plays a prominent role in pathogen defense by regulating immune activation and reinforcing structural barriers, often in coordination with JA and SA pathways to establish both local and systemic resistance. Nevertheless, certain pathogens can manipulate ET biosynthesis and signaling to promote infection, highlighting its dual role as both a defense regulator and a target of pathogenic manipulation [19].

## 3. ET Biosynthesis and Signal Transduction

### 3.1. Methionine-Derived ET Biosynthesis

Biosynthesis of ET occurs through a highly conserved enzymatic pathway originating from the amino acid methionine. In this pathway, methionine is first converted to S-adenosylmethionine (SAM) through an ATP-dependent reaction. SAM serves as a central metabolic intermediate and a universal methyl donor, representing a key junction between primary metabolic processes and phytohormone biosynthesis. SAM is metabolized by 1-aminocyclopropane-1-carboxylic acid synthase (ACS) to the ET precursor ACC. It constitutes the rate-limiting and principal regulatory step of ET production. This reaction also generates a by-product, 5-methylthioadenosine (MTA), which is recycled through the Yang cycle (methionine salvage cycle) to sustain methionine pools under prolonged stress conditions [39,40,41]. The Yang cycle involves a series of enzymatic steps that convert MTA back into methionine, thereby preventing depletion of methionine during continuous ET production. Key enzymes include methylthioadenosine nucleosidase (MTN), which hydrolyzes MTA to methylthioribose (MTR); methylthioribose kinase (MTK), which phosphorylates MTR to MTR-1-phosphate; and methylthioribose-1-phosphate isomerase (MTI), which rearranges this intermediate [41,42,43,44]. Downstream enzymes such as methylthioadenosine dioxygenase (MTO) and aminotransferases complete the regeneration of methionine, ensuring a closed metabolic loop [41]. This salvage pathway is critical because methionine is not only the precursor for ET but also an essential amino acid required for protein synthesis and methylation reactions. By maintaining methionine availability, the Yang cycle ensures sustained ET biosynthesis even under stress conditions where de novo methionine synthesis might be limited [40,41].

ACS is encoded by a multigene family whose isoforms are regulated at the transcriptional and post-translational levels via phosphorylation, ubiquitination, and proteasomal degradation. This allows flexible fine-tuning of ET biosynthesis in response to developmental and environmental stimuli [40,41,42]. Additionally, ACC itself functions as a signaling molecule that can undergo conjugation and be transported over long distances to mediate systemic responses [41,43].

The final step of ET biosynthesis is mediated by ACC oxidase (ACO), which converts ACC into ET, releasing carbon dioxide and hydrogen cyanide as by-products. The catalytic activity of ACO requires Fe (II) and ascorbate as essential cofactors, whereas molecular oxygen is required for the oxidation reaction, and CO_2_ serves as an activator. The binding of Fe (II) to the catalytic site of the enzyme is important to ensure the proper contact of ACC with the substrate. As a reducing agent, ascorbate triggers ring opening of the ACC intermediate via its catalytic activity [45].

AUX plays an important role in stimulating ET biosynthesis by enhancing the expression of ACS, which increases the availability of ACC, the immediate precursor of ET. In contrast, inhibitors such as aminoethoxyvinylglycine (AVG) reduce ET production by suppressing the activity of ACS. Similarly, cobalt ions inhibit ACO, limiting the final conversion of ACC to ET. Together, these regulatory mechanisms highlight the complex, multilayered control of ET biosynthesis through the modulation of key enzymatic activities and protein stability [40]. Overall, the ET pathway is an intricate metabolic feedback loop, incorporating enzyme activity, ACC transport, and hormonal crosstalk. This fine-tuning ensures that ET levels are dynamically controlled in plants to coordinate growth, development, and stress responses.

### 3.2. Perception and Signaling: ETR1, EIN2, EIN3, and ERF1

**Figure 2:**
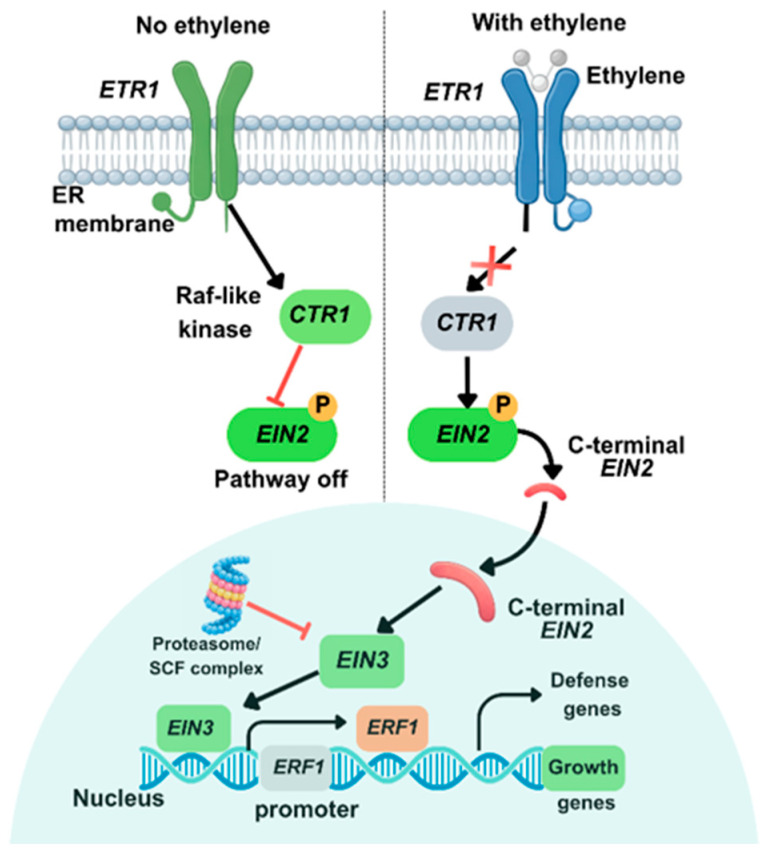
Schematic representation of the ET perception and signal transduction cascade at the ER membrane and nucleus. ET signaling in the absence (left) and presence (right) of the hormone. In the absence of ET, the receptor ETR1 activates Raf-like kinase CTR1, which inhibits downstream signaling via EIN2 phosphorylation. Upon ET binding, CTR1 is inactivated, leading to the cleavage and nuclear translocation of the EIN2 C-terminus. Inside the nucleus, the EIN3 transcription factor induces ERF1 expression, thereby modulating downstream transcription of specific growth- and defense-related genes. This model highlights the de-repression mechanism underlying ET-dependent responses.

ET perception and signaling are mediated by an interconnected signaling transduction network, consisting of key regulators such as ETR1, CTR1, EIN2, EIN3, and ERF1. ET is perceived at the endoplasmic reticulum membrane by receptor family members that structurally resemble bacterial two-component regulators and employ copper (Cu^+^) cofactors for ligand binding. In the absence of ET, ETR1 activates Raf-like kinase CTR1, which negatively regulates EIN2 by phosphorylation, thereby suppressing the ET response [46,47] (Figure 2). Upon ET binding, receptor activity is inhibited, leading to inactivation of CTR1 and release of its repression on EIN2 [48,49]. This enables proteolytic cleavage of EIN2 and translocation of its C-terminal fragment to the nucleus, where it initiates downstream signaling events [46,50]. Central to this nuclear response is the transcription factor EIN3. In the absence of ET, EIN3 is targeted for 26S proteasomal degradation by the SCF^EBF1/EBF2^ ubiquitin ligase complex. However, ET signaling stabilizes EIN3, allowing its accumulation and activation of early ET-responsive genes [50,51].

Among the downstream targets, ERF1, an ET response factor, plays a major role. It interacts with GCC box motifs in promoters of stress- and defense-related genes to mediate transcriptional reprogramming associated with growth–defense trade-offs [51]. Through this signaling cascade, ET functions as a central relay that links hormone perception to coordinated developmental and stress responses [46].

### 3.3. Feedback Regulation and Tissue-Specific Control

ET biosynthesis and signaling are subject to extensive feedback regulation and spatial control that allow plants to respond quickly to growth, ripening, or stress. ET can regulate the expression and activity of its own biosynthetic enzymes, forming autoregulatory loops that maintain hormonal homeostasis [41]. ACC further contributes to this regulation through its roles in transport and conjugation [41]. Tissue-specific dynamics add an additional layer of complexity. For example, in banana (*Musa* sp.), ET exhibits contrasting roles in distinct tissues, where it induces its own synthesis in the peel through induction of ACS and ACO expression while suppressing excessive production in the pulp, demonstrating coordinated regulation between adjacent tissues [52]. Small signaling molecules, such as H_2_S, can also modulate ET biosynthesis by inhibiting ACO activity and gene expression under stress conditions by posttranslational inhibition associated with oxidative signaling under osmotic stress in tomato plants [53].

ET signaling is further refined by feedback regulation that extends beyond primary transcriptional responses. Key transcription factors such as EIN3 and ERF1 not only mediate down-stream ET responses but also regulate the expression of genes involved in ET biosynthesis, thereby establishing autoregulatory circuits that tightly couple hormone production with signal transduction. Additional modulators, including proteins such as GDSL LIPASE1 (GDLP1), contribute to the dynamic regulation of these feedback loops by fine-tuning defense signaling through both activation and repression mechanisms [54].

Moreover, ET responsiveness is modulated through extensive crosstalk with other hormonal pathways, particularly JA, as well as environmental cues such as light. Photoreceptor-mediated signaling interacts with ETpathways at the transcriptional level, adjusting receptor sensitivity and downstream gene expression in a tissue- and developmental stage-specific manner [55]. This feedback and tissue-specific mechanisms together ensure that ET-mediated processes can operate with spatial specificity as well as temporal plasticity that is needed for the integration of plant growth, defense or stress tolerance.

### 3.4. Crosstalk with Key Hormones (JA, SA, ABA, AUX, and GA)

ET occupies a seminal position in phytohormonal crosstalk to integrate growth, defense, and adaptation. Its crosstalk with JA, SA, ABA, AUX, and GA influences growth–defense trade-offs. The ET-JA interaction is particularly critical for defense against necrotrophic pathogens such as *Botrytis cinerea*, where coordinated activation of ERF1 and *PDF1.2* drives effective immune responses, a process compromised in *ein2* or coi1 mutants [31] (Table 2), while ERF109 connects JA to AUX-modulated development [56]. In contrast, ET-SA interactions are context-dependent, often antagonistic in responses to biotrophic pathogens but capable of synergistic activation under combined stress conditions [57,58]. Similarly, ABA, primarily involved in abiotic stress tolerance, has shown dual responses by upregulation or downregulation of ET and JA-mediated responses under drought, salinity, and osmotic stresses [59]. Furthermore, AUX and ET crosstalk, mediated by reciprocal regulation of biosynthesis, transport, and perception, modulates growth plasticity upon root elongation and stress adaptation [60]. On the other hand, GA signaling is involved in crosstalk with ET via the GA-GID1-DELLA module, where DELLA proteins function as central hubs of growth and stress signaling coordination [11]. At the molecular level, these hormonal integrations also involve shared transcription factors, second messengers, or receptor modulation that form a hierarchical feedback cascade to influence the overall ET response in different tissues [60]. Thus, this crosstalk positions ET as a central coordinator within the phytohormonal network, functioning as a molecular integrator that fine-tunes growth, defense, and stress adaptation.

## 4. ET in Growth Regulation

### 4.1. Ethylene’s Role in Cell Expansion and Division

ET plays a multifaceted and context-dependent role in the regulation of cell division, acting in a tissue-specific manner and in response to both intrinsic developmental cues and external environmental signals. In some cases, ET promotes cell division. For instance, in the roots during early apical hook development and in root tissues, ET acts synergistically with AUX to stimulate cell division in subepidermal regions, a coordinated interaction that is essential for proper hook curvature [63]. Similarly, in *Marchantia polymorpha*, ET stimulates cell proliferation and expansion during gemmae development. ET-insensitive mutants develop embryos with fewer small cells, highlighting the hormone’s role in stimulation of meristematic growth [64]. In roots, ET acts in concert with AUX to regulate epidermal cell elongation and root hair initiation. This was demonstrated by adding exogenous AUX to rescue the response of the transport mutant indole-3-butyric acid-response (*ibr5*) or to alter its spatial distribution to recapitulate growth [65].

Exogenous retinaldehyde influences root growth by modulating endogenous AUX levels, indicating an interplay between peptides, hormones, and root development [66]. At the transcriptional level, members of the ERF-like NAC (ET response factor-like NAC) family of transcription factors, such as *RhNAC100* in rose petals, inhibit cell expansion, while their down-regulation promotes petal growth through increased expression of genes involved in modifying cell wall composition and aquaporins [67].

ET also interacts with BR to increase leaf hyponasty by modulating the local epidermal expansion process mediated by *ROTUNDIFOLIA3/CYP90C1*. Notably, BR-deficient mutants are impaired in ET-stimulated elongation [68]. Furthermore, ET regulates the cell cycle via stimulating DNA replication and endoreduplication while transiently inhibiting cytokinesis, as observed in cucumber hypocotyl epidermal cells stalled at a G2-like stage [69]. The ET precursor ACC has independent functions in regulating cell wall remodeling and GMCD [43,64]. Taken together, ET is a versatile regulator that integrates transcriptional networks, hormonal crosstalk, and the cell cycle to coordinate growth with environmental adaptation.

### 4.2. Regulation of Root and Shoot Architecture

**Figure 3:**
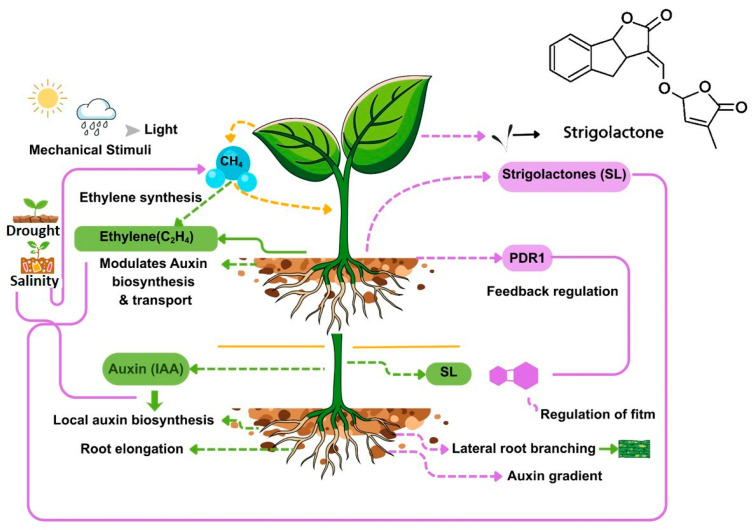
Synergistic regulation of root and shoot architecture by the ET-AUX -SL signaling network. The diagram illustrates how environmental stimuli, such as light, mechanical stress, drought, and salinity, stimulate ET biosynthesis to modulate plant development. ET modulates auxin (IAA) biosynthesis and transport, which regulates root elongation and lateral root branching. Interactions with strigolactones (SLs), including feedback regulation via PDR1, further refine root and shoot architecture in response to environmental fluctuations.

ET is a classic apical hormone that influences plant architecture and interacts with other phytohormones to influence both root and shoot growth. In roots, ET contributes to developmental plasticity by modulating primary and lateral root responses to nutrient availability and stress conditions [70]. ET regulates root gravitropism and root angle in cereals by modulating AUX biosynthesis and the AUX-dependent gravitropism machinery; ET-insensitive mutants show altered AUX levels and impaired root angle control [71]. Under drought, salinity stress, or nutrient limitation, ET-driven alterations in AUX gradients regulate lateral root emergence and elongation, thereby optimizing resource acquisition and stress tolerance [70]. In aerial tissues, ET regulates shoot architecture, including branching patterns, leaf morphology, and meristem activity, acting as a molecular integrator of mechanical and light signals. Its crosstalk with SL provides an additional layer of regulatory complexity. SL stimulates primary and adventitious root growth but also inhibits lateral root formation by 30–50%. SL interacts with AUX to influence growth patterning and resource allocation [72,73,74] (Figure 3). Regulation of SL transporters by PDR1 facilitates root-to-shoot hormonal crosstalk that couples nutrient acquisition to branch and canopy development in order to adjust C-N balance in diverse soil environments [75].

### 4.3. Influence on Senescence, Flowering, and Fruit Ripening

ET plays a central role in regulating key developmental transitions, including senescence, flowering, and fruit ripening, by integrating genetic programs with environmental signals. During leaf senescence, ET promotes the expression of senescence-associated genes (SAGs) and accelerates chlorophyll degradation, whereas reduced ET signaling delays senescence and prolongs leaf longevity [76]. During reproductive development, ET regulates processes such as flowering initiation, grain filling, and seed development in crops like rice [77]. These processes are modulated by hormonal crosstalk of ET with AUX, GA, and ABA, connecting reproductive development with nutrient availability and stress response.

In climacteric fruits, ET serves as a key regulator of ripening by stimulating respiration and autocatalytic ET production through the coordinated activity of ACS and ACO enzymes. Inhibition of these enzymes reduces ET activity, resulting in delayed color change and softening, thus extending fruit life span [78]. ET is also rapidly induced in response to wounding and environmental stress, primarily through the upregulation of ACS expression [79]. In tomato, multiple ET receptors like ETR1, ETR3, ETR6, and ETR7 regulate ET signal transduction during fruit ripening [80]. Thus, ET serves as a master regulatory molecule that integrates developmental transitions and stress responses to maximize plant fitness, productivity, and postharvest longevity.

## 5. ET in Plant Defense Responses

### 5.1. Activation of Defense-Related Transcription Factors

ET is a central regulator of plant immunity, modulating defense responses through the activation of key transcription factors, including ET response factors (ERFs) and WRKY proteins, which collectively regulate the expression of defense-related genes. Members of the AP2/ERF family play a critical role in growth adaptation to pathogen attack, hormonal signaling, secondary metabolism, and cell wall fortification involving both MAPK-dependent and -independent pathways [81]. ERFs mediate the crosstalk between ET and JA through interaction with GCC-box cis-elements in the promoters of defense genes and activate transcriptional reprogramming that enhances host resistance to pathogen infection. Key members such as ERF1, ORA59, and ERF6 and 96 act as positive regulators of ET-mediated immunity [82].

Functional diversification within the ERF family enables fine-tuned regulation of defense intensity and specificity. For example, OsAP2/ERF152 integrates SA and JA/ET signaling pathways to confer broad-spectrum resistance against bacterial and fungal pathogens [83] (Table 3). Similarly, AtERF2 enhances resistance to necrotrophic pathogens, whereas AtERF4 modulates the expression of JA-responsive genes to balance immunity and growth [84]. Additionally, AtERF14 is a master regulator of defense genes. Mutants deficient in AtERF14 exhibit impaired defense activation and increased susceptibility to pathogens [85]. ET-inducible factors such as TERF1 contribute to oxidative stress tolerance by regulating reactive oxygen species (ROS) scavenging systems [86].

WRKY transcription factors govern ET-induced defense signaling by modulating gene expression and hormone crosstalk. They are rapidly upregulated at the early stage of infection and serve as positive regulators of immune responses to various biotic stresses. A large number of WRKY genes are transcriptionally modulated by ET and other defense hormones, suggesting their integrative action in ET-mediated immune response [23,87]. Functionally, WRKY genes control transcriptional cascades and can directly interact with pathogen effectors or act as decoys, playing a role in modulating the dynamics of plant–pathogen interactions [87]. The activities of ERFs and WRKYs are precisely regulated at the transcriptional, post-transcriptional, and post-translational levels to balance the growth–defense trade-off [88]. For example, ERF1 is phosphorylated by mitogen-activated protein kinases (MAPKs), which enhances its stability and transcriptional activity under stress conditions [89,90]. Similarly, WRKY33 is subject to ubiquitin-mediated proteasomal degradation, with E3 ligases such as RGLG2 modulating its abundance to fine-tune defense responses [91,92]. Post-transcriptional regulation also occurs via microRNAs, such as miR396, which target WRKY transcripts to adjust their expression during pathogen attack [93]. As central regulatory hubs, ERFs mediate ET, ABA, and JA signaling pathways to integrate biotic and abiotic stress responses [8].

**Table 3:** Systematic overview of stress-responsive transcription factors, their target genes, and resulting phenotypic outcomes in crop and model species.

Transcription Factors	Family/Subgroup	Regulated Gene(s)/Pathway	Stress Type/Inducer	Physiological/Functional Outcomes	References
*OsAP2/ERF152*	AP2/ERF	SA and JA/ET-responsive defense genes	Bacterial and fungal pathogens	Enhances resistance to bacterial and fungal infections	[83]
*SlERF-B3*	ERF	TYLCV-responsive	Tomato yellow leaf curl virus	Differentially expressed in resistance	[83]
*JERF1*	AP2/ERF (ERF subfamily)	*OsP5CS* → proline biosynthesis	Drought	Higher proline + stress-responsive genes → improved drought tolerance (rice).	[23]
*JrWRKY7*	WRKY family	Works with *JrERF2-2* to regulate GSTs	Drought	Improved drought tolerance (walnut) (through GST regulation).	[23]
*GmNAC12*	NAC	Responsive to ABA and ET signaling interactions	Drought; ABA; ET	Enhances drought stress tolerance (upregulated under drought/ABA/ET)	[94]
*TaDTG6-B*	DREB TF	Broad transcriptional regulatory networks	Drought	Gain of function improves drought tolerance in seedlings	[94]
*ZmWRKY20* (with *ZmWRKY115*)	WRKY	*ZmWRKY20*-*ZmWRKY115* repress *ZmbZIP111* by binding its promoter	Salt stress	Higher salt tolerance and lower ROS in the *zmwrky20* mutant vs. WT	[95]
*SlWRKY57*	WRKY TF family	Directly attenuates *SlRD29B*, *SlDREB2*, and ion homeostasis gene *SlSOS1*	Salt stress	Negative regulator of salt-stress response	[96]
*SlWRKY81*	WRKY	H_2_O_2_ mediated stomatal closure genes	Drought	Negative regulation of stomatal closure	[97]
*SlERF1*	AP2/ERF (ERF)	Defense genes *PAL1*, *NPR1*, *PR1*; ROS scavenging genes *RBOHD*, *CAT*, *SOD*, *LOX1*	*Phytophthora capsici* infection (tomato)	ISR activation with stronger defense + ROS scavenging.	[98]

### 5.2. Ethylene’s Role in Induced Systemic Resistance (ISR)

GLIP1 mediates ET-induced systemic resistance by regulating key ET signaling components, such as EIN3 and ERF1, to confer broad-spectrum pathogen resistance [99]. The plant growth-promoting rhizobacterium *Bacillus cereus* AR156 has been shown to induce systemic resistance (ISR) in *Arabidopsis* by activating both SA- and JA/ET-dependent signaling pathways. Notably, *etr1* mutants exhibit reduced protection, underscoring the essential role of ET perception in the defense response [100]. Moreover, biocontrol bacteria-ISR is mediated through an ET-dependent pathway that is distinct from the accumulation of SA and the expression of pathogenesis-related genes [101]. AR156-ISR relies on JA and ET signaling via an NPR1-dependent pathway, as supported by reduced resistance in *jar1*, *ein2*, and *npr1* mutants [102].

ISR involves a coordinated interaction between the JA and ET signaling pathways. During beneficial microbe interactions, plants often show increased expression of ET biosynthesis and ET-responsive genes, indicating a complex regulatory network that may also include small RNAs (sRNAs) [103]. *GLIP1* mutants are defective in ISR, pinpointing ET-associated molecular regulation as critical for systemic immunity induced by necrotrophic pathogens [99].

### 5.3. Modulation of Programmed Cell Death (PCD) and Hypersensitive Response (HR)

ET is a key regulator of programmed cell death (PCD) and hypersensitive response (HR), which restricts pathogen spread via localized cell death. ET is involved in the regulation of HR and defense gene activation along with SA. ET signaling components, including EIN2 and EIN3, are required for the execution of full HR development and subsequent formation of lesions [104]. ET regulates PCD by regulating the transduction of defense-responsive genes, proteases, and nucleases for waterproofing cells. Additionally, ET-dependent autophagy contributes to immune regulation. For example, autophagic factor RabG3b promotes vacuolar degradation during HR in an ET-dependent manner, linking hormone signaling to immune cell death [105]. γ-Aminobutyric acid (GABA) alleviates programmed cell death by reducing caspase-3-like activity under cadmium stress, suggesting that ET may act conversely to promote such protease activities during PCD [106]. Furthermore, ET induces ROS accumulation and antioxidant enzyme activities in response to pathogen attack, thereby modulating cell death associated with HR [107]. It is also involved in the regulation of cytoskeletal reorganization and vacuolar rupture, structural changes that are important for cell degradation in HR [108]. Environmental factors often interact with ET signaling under hypoxic conditions and HR-driven PCD is blocked, while defense-associated gene expression is maintained; this suggests that ET-signaled death and defense can be separated for effective, energy-saving immune regulation [109].

### 5.4. Pathogen Exploitation and Suppression of ET Signaling

Pathogenic microbes have evolved complex strategies to manipulate ET biosynthesis, perception, and downstream signaling to suppress host defenses and enhance virulence. ET, together with JA and SA, regulates genes essential for defense responses against necrotrophic and hemibiotrophic pathogens [56]. ET biosynthesis and signaling also feedback on JA-responsive gene expression. External treatments such as acetylsalicylic acid modulate ET biosynthetic genes and MAPK activities post-transcriptionally, thereby influencing the activation status of JA/ET-regulated defense genes [110]. However, pathogens often exploit antagonistic mechanisms such as immune evasion strategies, stress responses, and effector proteins to breach host-specific barriers and establish infections [111]. While JA-ET signaling synergistically enhances defense against necrotrophs, SA can antagonize this response, creating vulnerabilities that pathogens can exploit [56]. β-Aminobutyric acid (BABA)-induced resistance triggers extensive phytohormone signaling involving ABA, JA, SA, and ET. Differential transcription factor regulation in *Arabidopsis* suggests antagonistic and synergistic effects, including modulation of *MYC2* that links JA signaling with other hormone pathways [112]. Mutants such as *ein1*, *ein2*, *ein3* and *eil1A* that fail to transduce ET signaling are more susceptible to pathogen attack than the wild type [113]. Pathogen-mediated interference also extends to the modulation of ROS production and hypersensitive cell death, thereby weakening the plant’s ability to contain infections. These phenomena indicate that pathogens can disrupt ET signaling at multiple levels, from biosynthesis to receptor interaction and hormonal crosstalk, to suppress plant defense. Strengthening ET signaling could, therefore, enhance plant immunity while maintaining a balance between growth and defense under pathogen attack.

## 6. ET as a Molecular Coordinator of the Growth–Defense Balance

### 6.1. Integrative Signaling: Balancing Growth Suppression and Defense Activation

ET functions as a master integrator of growth inhibition with defense activation under fluctuating environmental conditions. By redirecting metabolic resources toward defense, ET suppresses organ elongation, particularly through regulation of epidermal cell expansion, and coordinates developmental reprogramming in close interaction with AUX. Signal transduction through core components such as ETR1, EIN2, and EIN3 links perception of ET at the ER with nuclear transcriptional regulation [9,114].

ET acts through crosstalk, particularly with JA and SA, which enables context-dependent modulation of immune responses. The cooperative interaction between ET and JA under biotic stress promotes resistance against necrotrophic pathogens and herbivores, often accompanied by suppression of SA-mediated pathways [115]. The synergistic ET-JA module, mediated through transcriptional regulators such as EIN3 and ORA59, drives the expression of defense genes, including *PDF1.2*, conferring resistance to necrotrophic pathogens [116]. In contrast, ET-SA interactions are often antagonistic, reflecting their specialization in defense against necrotrophic and biotrophic pathogens, respectively. ET can suppress SA-mediated immunity, for example, through EIN3-dependent modulation of ORA59 stability, thereby prioritizing appropriate defense pathways. However, this antagonism is not absolute; synergistic interactions between ET and SA have been reported under specific combined stress conditions, highlighting the context-dependent nature of hormonal integration [56,57,116]. ET signaling is also modulated through interactions with ABA, particularly in responses to abiotic stress. While ET and ABA often act antagonistically, such as in the regulation of stomatal closure during drought, cooperative interactions can occur depending on the stress context. For example, ABA may enhance JA-mediated resistance to herbivores, while under flooding conditions, it can suppress JA responses, thereby reshaping defense priorities [10,61,117]. This hormonal plasticity allows ET to balance between defense and growth responses.

At the transcriptional level, factors such as ERF6 function in this integrative regulation by simultaneously activating defense-related genes while repressing growth-associated processes, thereby optimizing resource allocation during stress [29]. Additionally, ET is involved in regulating epigenetic mechanisms. Histone acetylation and other chromatin modifications enhance the accessibility of ET-responsive genes, favoring sustained defense gene expressions under prolonged stress conditions [38]. At the subcellular level, dynamic relocalization of signaling components further refines ET responses. For example, CTR1 is canonically described as a Raf-like kinase that suppresses ET signaling at the ER by phosphorylating EIN2. Upon ET perception, CTR1 activity is inhibited, leading to EIN2 activation and stabilization of EIN3 [46,48]. Recent findings, however, reveal that CTR1 can also relocalize to the nucleus under ET treatment [118]. Rather than contradicting the canonical model, this nuclear relocalization represents a non-canonical regulatory layer, where CTR1 interacts with components of the EIN3 degradation machinery to fine-tune EIN3 stability and extend defense signaling duration. This nuanced mechanism highlights additional complexity in ET regulation, ensuring continuous defense under stress while potentially delaying growth rebound.

### 6.2. Temporal and Spatial Tuning of ET Signaling

Endogenous ET signaling is strictly controlled both spatially and temporally to coordinate growth and defense. Temporal regulation is achieved through transcriptional cascades governed by master regulators such as EIN3, which coordinate early growth inhibition with subsequent activation of defense programs [119]. Spatial regulation further fine-tunes ET responses in a tissue- and cell-specific manner. For instance, the epidermis serves as an important site for ET-mediated growth repression, while differential distribution of receptors and signaling components across tissues establishes tissue-specific response thresholds, preventing systemic repression of growth [114]. ET signaling is also partitioned across distinct plant tissues, enabling context-dependent regulation of growth and defense outcomes. In roots, ET interacts closely with AUXto regulate root elongation, gravitropism, root angle, and lateral root development, thereby optimizing nutrient acquisition and adaptation to environmental stresses [60,71,120]. In shoots, ET modulates branching patterns, leaf architecture, and meristem activity through crosstalk with AUX, GA, and SL, balancing vegetative growth with defense requirements [121,122,123]. Vascular tissues contribute to systemic signaling through the transport of ACC, the immediate precursor of ET, facilitating long-distance communication between roots and shoots during stress responses. In reproductive organs, ET regulates flowering, senescence, fruit ripening, grain filling, and seed development, integrating developmental processes with environmental cues [124,125,126]. Together, these tissue-specific signaling modules enable ET to fine-tune resource allocation and coordinate growth–defense trade-offs at the whole-plant level.

Subcellular dynamics, including the nuclear relocalization of CTR1, add another layer of spatial control, allowing precise modulation of signaling duration and intensity. Upon ET sensing, CTR1 is transported from the ER to the nucleus, where it stabilizes EIN3 and prolongs the ET response, playing a role in regulating growth-to-defense transition times, allowing it to be adjusted according to external environmental advancement [118]. In summary, precise temporal and spatial regulation of ET signaling spanning receptor sensitivity, transcription factor activity, subcellular trafficking, chromatin remodeling, and hormonal feedback ensures optimal growth suppression and defense activation.

## 7. Stress-Specific Ethylene Responses

### 7.1. Abiotic Stress

ET is a central, stress-responsive hormone that mediates plant resilience to abiotic stressors, including drought, salinity, flooding, and temperature. Under drought conditions, ET biosynthesis and signaling are rapidly induced and interact with ABA to regulate stomatal closure and minimize water loss. The transcription factor ERF1 coordinates gene networks associated with water use efficiency and antioxidant defense, resulting in a reduction in water loss and the induction of stress-responsive genes [127].

**Figure 4:**
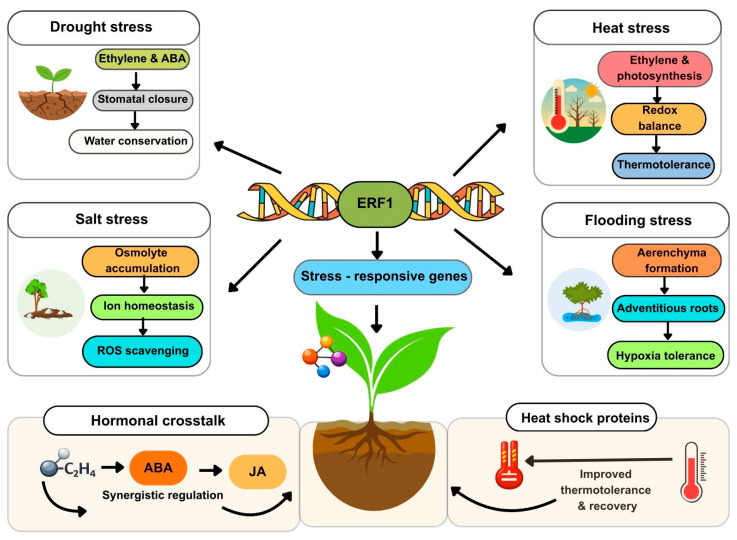
ET-mediated physiological and molecular adaptive strategies against abiotic stressors. The diagram highlights the central role of ERF1 in orchestrating stress-specific responses, including stomatal closure for drought, ion homeostasis for salinity, thermotolerance for heat, and aerenchyma formation for flooding. Key metabolic and morphological responses, such as ROS scavenging, osmolyte accumulation, and adventitious root development, are depicted, which collectively enhance plant resilience. Synergistic crosstalk among ET, ABA, and JA in regulating downstream stress-responsive genes and heat shock proteins underscores the plasticity of ET signaling in maintaining redox balance and physiological stability under adverse environmental conditions.

During high salinity, ET mitigates osmotic stress by promoting the accumulation of osmolytes (e.g., proline), regulating ion homeostasis, and enhancing antioxidant enzyme activity to alleviate salt-induced oxidative damage (Figure 4). Members of the ERF1 family bind to GCC or DRE/CRT binding elements in defense gene promoters, thereby promoting ion accumulation and scavenging ROS. Hormonal crosstalk among ET, ABA, and JA plays a role in these responses to preserve cellular homeostasis under salinity [59,127].

Flooding triggers ET production due to restricted gas diffusion, triggering morphophysiological rearrangements like aerenchyma and adventitious rooting that restore oxygen balance. The maize ET-responsive ERF protein ZmEREB180 enhances hypoxia tolerance in *Arabidopsis* and maize through modifications in root system architecture and nitrogen metabolism [128]. Similar ET-mediated responses have been reported in banana and *Arabidopsis*, suggesting a conserved role of this hormone during anaerobic stress tolerance [129,130,131].

ET also acts systemically to coordinate whole-plant adaptation. In soybean, ET and ABA jointly reprogram carbohydrate metabolism during submergence [132]. ET also activates heat shock proteins under elevated temperatures and, together with other factors, selectively enhances thermotolerance and recovery [133].

### 7.2. Biotic Stress

ET is a key signaling hormone that mediates defense against biotic stresses like fungi, bacteria, and herbivores. These responses are regulated by ERFs from the APETALA2/ERF family, which regulate ET-dependent gene expression by interacting with cis-acting elements such as GCC boxes and interact with phytohormones like JA and SA to modulate immunity [8,134]. During plant–fungus interactions, ET biosynthesis and signaling pathways are induced and involved in the activation of PR genes, biosynthesis and accumulation of antifungal secondary metabolites, cell wall strengthening, and ROS formation. For instance, the pepper extracellular peroxidase CaPO2, which is partly ET-regulated, plays a significant role in resistance to *A. brassicicola*, a fungal pathogen [135]. Similarly, meta-analyses in apple highlight the central role of ET signaling, with ERF and WRKY transcription factors acting as hubs in coordinated immune networks [136].

ET plays context-dependent roles in resistance against bacterial diseases. Pre-treatment with chloride salts strengthens broad resistance via ET and SA pathways and suppresses *Pseudomonas syringae* growth [137]. While some pathogens can exploit ET signaling to induce host susceptibility, plants activate an ET-dependent defense response network that interacts with JA and SA signaling pathways to defend themselves [56,57]. Beneficial endophytes reprogram ET response pathways to support positive effects on plant growth and resistance to fungal and bacterial pathogens [138,139].

Herbivore attack induces ET synthesis, which acts synergistically with JA signaling to promote the expression of defense proteins and secondary metabolites, thereby strengthening plant resistance to insect herbivory [56,140]. Sophisticated data-driven feature engineering pipelines integrating multi-dimensional omics datasets enable the robust identification of key drought-tolerant genes in soybean. These pipelines exemplify the scalability and accuracy of multi-omics approaches that could be extended to uncover ET-responsive genes modulating biotic stress resistance [141].

### 7.3. Combined and Sequential Stresses

**Figure 5:**
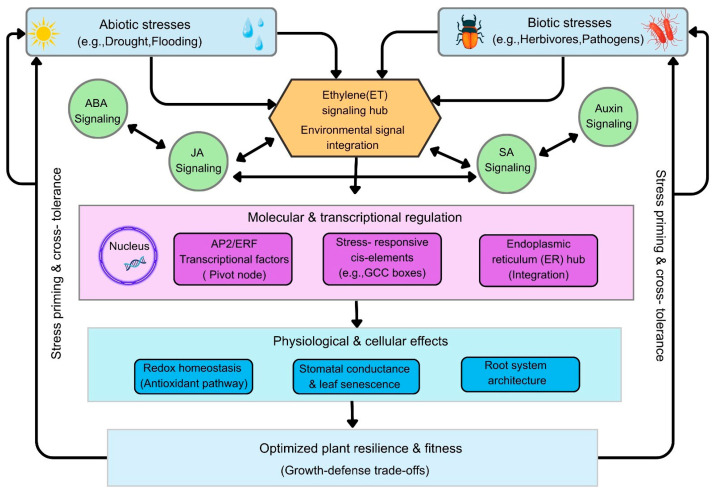
Integration of combined abiotic and biotic stress signals through ET signaling. ET serves as a central signaling hub that interacts with the ABA, JA, AUXIN, and SA pathways in response to simultaneous environmental challenges. This convergence activates AP2/ERF transcription factors and stress-responsive cis-elements to regulate the downstream molecular machinery in the ER and nucleus. The resulting physiological adjustments, ranging from redox homeostasis to altered root architecture, optimize the trade-off between growth and defense. Feedback mechanisms involving stress priming further enhance long-term plant resilience and fitness under complex environmental conditions.

Plants face combined abiotic and biotic stresses that require complex signaling to balance growth, defense, and fitness. ET functions as a central hub in these scenarios, integrating environmental cues with hormonal and transcriptional responses to optimize plant fitness. Through interactions with ABA, JA, and SA, ET mediates cross-tolerance mechanisms. For example, JA-ABA interactions may enhance resistance under combined drought and herbivory, whereas flooding conditions can suppress JA-dependent defenses, reflecting a shift in resource allocation priorities [117,142] (Figure 5). Under combined drought and heat stress, ET further contributes to the maintenance of photosynthetic efficiency and redox homeostasis [133].

At the molecular level, the interaction between abiotic and biotic stresses triggers a transcriptome and hormonal regulation that are distinct from those caused by individual stress. The APETALA2/ETHYLENE RESPONSE FACTOR (AP2/ERF) family forms a pivotal node of this integration since they selectively bind through their conserved APETALA2 domains stress-responsive cis-regulatory elements and induce stress-specific activation of defense and acclimation-related genes. ET also contributes to the regulation of redox homeostasis under multiple stresses, often inducing antioxidant pathways that stabilize cellular function. Studies in sunflower and other species have demonstrated overlapping activation of antioxidant defenses under concurrent abiotic and biotic stresses, highlighting ET’s role in maintaining cellular integrity [143,144]. Moreover, ET contributes to stress “priming”, whereby prior exposure to stress enhances tolerance to subsequent challenges through sustained transcriptional reprogramming and cross-tolerance mechanisms [145]. Developmental context further shapes these responses, as age-dependent hormonal sensitivity influences how plants allocate resources between growth, defense, and stress tolerance [142]. Overall, ET acts as a molecular hub coordinating growth–defense trade-offs under combined stresses by mediating the hormonal, transcriptomic, and physiological output that optimizes plant resilience to fluctuating environments.

## 8. Future Perspectives and Knowledge Gap

Despite substantial advances in elucidating the role of ET in regulating the growth–defense balance, several key gaps remain that limit its full translation into agricultural applications. One key area is understanding nuclear mechanisms linking ET signaling to chromatin modification. Recent studies have shown that nuclear EIN2-C interacts with the pyruvate dehydrogenase complex (PDC) for acetyl-CoA production, which is essential for histone acetylation. However, the epigenetic mechanisms and their role in development and stress responses remain unclear [146].

The tissue specificity of ET regulation remains another unresolved area. Although the epidermis is recognized as a scaffold for EIN3 degradation, it is unknown whether other tissues, notably roots, shoots, or reproductive structures, contribute to ET-induced growth–defense trade-offs [114]. Addressing this spatial variability is essential for understanding how ET signaling is differentially deployed across plant organs under stress.

At the transcriptional level, the functional diversification and regulatory hierarchies within the EIN3/EIL1 regulon remain incompletely characterized. In particular, the role of ERF6 in mediating stress-induced growth repression requires further investigation, as do the mechanisms governing ET-independent activation pathways and interactions with other transcription factor networks [147]. Similarly, although extensive crosstalk between ET and other phytohormones such as SA, JA, and ABA has been documented, a fundamental question persists: how do plants optimize these interactions to maximize defense while minimizing fitness costs [148]? Moreover, the regulation of ET signaling during host–pathogen interaction, particularly when and how environmental cues tune ET emission, is only partly understood [19].

Emerging technologies such as single-cell transcriptomics, biosensors, and machine learning (ML) provide promising tools to address these challenges. Single-cell transcriptomics now enables the dissection of gene expression at cellular resolution, offering unprecedented insights into tissue-specific signaling and stress heterogeneity [149,150]. Additionally, biosensors allow real-time monitoring of physiological parameters, facilitating integration of molecular and phenotypic data for precision agriculture [151]. The application of ML approaches further enhances the capacity to integrate multi-omics datasets and predict plant responses to complex environmental stimuli [151]. Recent advances highlight that combining single-cell/nucleus RNA sequencing with spatial transcriptomics can resolve cell-type-specific transcriptional responses to both biotic and abiotic stresses, bridging the spatial and temporal dimensions of stress biology [152]. Such approaches directly address the tissue-specificity gap in ET signaling by mapping heterogeneity across cell types and developmental stages, while integration with ET biosensor lines adds a real-time physiological dimension. Thus, leveraging next-generation technologies alongside mechanistic insights will enable the optimization of ET pathways, ultimately supporting the development of resilient crops with minimized fitness trade-offs and advancing sustainable agricultural systems.

## 9. Conclusions

ET is one of the key molecular orchestrators of the plant growth–defense trade-off, connecting developmental programs with biotic and abiotic stress signaling pathways so that plants can enhance their overall fitness under varying environmental conditions. Rather than a simple growth inhibitor or stress hormone, ET is now considered a context-dependent decision-making signal that redirects metabolic and energetic resources in an environmentally responsive manner. With a well-defined biosynthetic and signaling cascade, as well as significant crosstalk with other hormonal pathways, ET fine-tunes growth restraint to immune activation and stress acclimation in a very plastic way. Importantly, ET plays a multifaceted role in plant immunity and resilience, coordinating defense and adaptive responses against a broad spectrum of pathogens, herbivores, and abiotic stresses through its interactions with JA, SA, ABA, ROS, and other signaling networks. By integrating developmental cues with immune signaling pathways, ET enables plants to dynamically prioritize defense activation while minimizing detrimental impacts on growth, thereby contributing to both disease resistance and overall plant fitness.

From here forward, it will be crucial to incorporate multi-omics strategies, spatial and single-cell analyses, and dynamic stress modeling to dissect how ET signaling functions throughout developmental stages and environmental scenarios. Mechanistic insights can be gained through targeted genetic modification of ET sensitivity, signaling components, or hormonal crosstalk, thereby supporting the development of climate-resilient crops with a balanced growth–defense trade-off. By explicitly linking immunity and abiotic stress tolerance, ET emerges as a unifying molecular coordinator for sustainable crop productivity under climate change.

## Figures and Tables

**Table 2:** Integration of ET signaling with diverse hormonal pathways to modulate stress-specific physiological and molecular responses.

Interacting Hormones	Stress Type	Mechanistic Interaction with ET	Physiological/Molecular Outcome	References
JA	Necrotrophic fungal pathogens (e.g., *Botrytis cinerea*, *Alternaria brassicicola*)	JA and ET act synergistically: in *ein2* (ET signaling) or *coi1* (JA signaling) mutants, JA + ET cannot induce key defense genes (e.g., *ERF1*, *PDF1.2*)	Activation of JA/ET defense gene programs (*ERF1*, *ORA59*, *PDF1.2*) leading to enhanced defense against necrotrophs	[31]
SA	(Hemi)biotrophic vs. necrotrophic pathogen trade-off, incl. *Pseudomonas syringae* (hemi-biotroph) vs. *B. cinerea* (necrotroph)	JA-ET-SA network where JA + ET promote necrotroph defense, while SA promotes biotroph/hemi-biotroph defense	Defense allocation/trade-offs: biasing toward SA/PAL supports (hemi)biotroph defense, while biasing toward *WRKY33* → JA/ET supports necrotroph defense	[31]
ABA	Abiotic (water stress/drought)	Proposed via *ABI4* repression of *ACS4/ACS8*	Lower ET biosynthesis under water-stress-associated high ABA (via ACS repression)	[61]
	Abiotic (UV-B stress)	ET proposed as an activator of ABA synthesis under UV-B but high ET blocks ABA accumulation, while ABA inhibits UV-B-induced ET production	Under UV-B: ABA rises (~3-fold) as ET peaks early then declines	[61]
AUX (IAA)	Abiotic (drought; endophytes/PGPR with ACC deaminase)	Under drought, inoculation associated with reduced ET and higher IAA levels (linked to ACC deaminase lowering ET).	Higher IAA and improved plant physiological performance vs. non-inoculated controls under drought.	[14]
JA (with ACC/ET precursor context)	Biotic (ectomycorrhizal interaction stage)	In *Laccaria bicolor Populus* interaction, ACC and JA application repress Hartig net formation; ET and JA regulated genes appear in late interaction stages.	Reduced Hartig net formation (secondary colonization stage) and altered expression of cell wall biosynthesis/maintenance genes.	[14]
CK	Biotic (disease/pathogen response)	*CRF5* (CK response factor; *AP2/EREBP* family) links pathogen response with CK signaling within ET-responsive TF networks	Supports disease resistance signaling connection (pathogen response ↔ CK signaling)	[23]
Nitrous oxide (NO)	Abiotic (postharvest/ripening physiology)	NO-ET crosstalk via partial inhibition of ET biosynthesis: reduced ACS/ACO activity, downregulation of *SlACS2/4* and *SlACO1/3* (also *CDPK/MAPK* effects mentioned)	Reduced ET release and delayed tomato ripening (breaker stage delay)	[23]
Dopamine	Abiotic (salinity)	Exogenous dopamine (apple) induces ET formation and inhibits oxidation of IAA under salinity stress.	increased ET formation + reduced IAA oxidation (shift in ET-AUX balance) under salinity.	[62]
GA	Abiotic (flooding/low oxygen)	During flooding, ET accumulation leads to increased GA under low-oxygen conditions.	Supports an adaptive response under flooding stress (via GA increase following ET build-up).	[62]
BRs	Abiotic (salt stress)	ET is reported as a major component in the BR-induced alternative respiratory pathway in cucumber seedlings under salt stress.	Alternative respiratory pathway activation in the salt-stress response context.	[62]

## Data Availability

No new data were created or analyzed in this study. Data sharing is not applicable to this article.
